# Biomarkers for predicting efficacy of PD-1/PD-L1 inhibitors

**DOI:** 10.1186/s12943-018-0864-3

**Published:** 2018-08-23

**Authors:** Ming Yi, Dechao Jiao, Hanxiao Xu, Qian Liu, Weiheng Zhao, Xinwei Han, Kongming Wu

**Affiliations:** 1 Department of Oncology, Tongji Hospital of Tongji Medical College, Huazhong University of Science and Technology, Wuhan, 430030 China; 2 Department of Interventional Radiology, The First Affiliated Hospital of Zhengzhou University, Zhengzhou, 450052 China

**Keywords:** PD-1/PD-L1 inhibitors, Predictive biomarkers, Tumor mutational burden, Microsatellite instability, Gut microbiota, Peripheral biomarker

## Abstract

Programmed cell death protein 1/programmed cell death ligand 1 (PD-1/PD-L1) is a negative modulatory signaling pathway for activation of T cell. It is acknowledged that PD-1/PD-L1 axis plays a crucial role in the progression of tumor by altering status of immune surveillance. As one of the most promising immune therapy strategies, PD-1/PD-L1 inhibitor is a breakthrough for the therapy of some refractory tumors. However, response rate of PD-1/PD-L1 inhibitors in overall patients is unsatisfactory, which limits the application in clinical practice. Therefore, biomarkers which could effectively predict the efficacy of PD-1/PD-L1 inhibitors are crucial for patient selection. Biomarkers reflecting tumor immune microenvironment and tumor cell intrinsic features, such as PD-L1 expression, density of tumor infiltrating lymphocyte (TIL), tumor mutational burden, and mismatch-repair (MMR) deficiency, have been noticed to associate with treatment effect of anti-PD-1/anti-PD-L1 therapy. Furthermore, gut microbiota, circulating biomarkers, and patient previous history have been found as valuable predictors as well. Therefore establishing a comprehensive assessment framework involving multiple biomarkers would be meaningful to interrogate tumor immune landscape and select sensitive patients.

## Background

Novel cancer immunotherapy is the most promising cancer treatment strategy, mainly including chimeric antigen receptor T cell, bispecific antibodies and immune checkpoint inhibitors [1–4]. Programmed cell death protein 1/programmed cell death-ligand 1 (PD-1/PD-L1) axis is a vital immune checkpoint signaling pathway which could downregulate magnitude of inflammation response and maintain immune homeostasis [5]. Immune receptor tyrosine based inhibitory motif (ITIM) and immune receptor tyrosine-based switch motif (ITSM) are core structures of PD-1, which transduct extracellular signal and recruit Src homology 2 domain containing phosphatases 1/2 (SHP1/2) within the cell [6]. PD-1/PD-L1 axis impairs activation of T cell by inhibiting Ras-Raf-MEK-ERK and PI3K-AKT signaling pathways which are generally believed to promote proliferation and differentiation of T cell [7]. The inhibitory regulation of PD-1/PD-L1 is usually compared to a brake for activation of T cell [8].

In the evolution of immunity, PD-1/PD-L1 axis is an indispensable pathway to maintain immune tolerance and prevent autoimmunity diseases [9–11]. However, PD-1/PD-L1 axis influence the balance between tumor immune surveillance and immune resistance as well [12, 13]. Elevated PD-L1 expression on tumor cell or tumor infiltrating lymphocyte (TIL) results in the exhaustion of T cell [14], thus the attenuated tumor-specific immunity promoting tumor progression [15].

Based on the mechanism mentioned above, PD-1/PD-L1 inhibitors block the negative regulatory signal pathways and unleash T cell from exhausted status [16]. Since first PD-1/PD-L1 inhibitor (pembrolizumab) was approved by Food and Drug Administration in 2014, many immune checkpoint inhibitors have been applied in clinical practice [17, 18]. PD-1/PD-L1 inhibitors show potent and durable anti-tumor effects, especially in some refractory tumors [4, 19, 20]. Even though the relatively low response rate limits the application in patients, PD-1/PD-L1 inhibitors attract extensive attention [21–23].

In clinical practice, the primary problem for application of PD-1/PD-L1 inhibitors is the unsatisfactory response rate in overall patients. Therefore, patient selection should be implemented prior to PD-1/PD-L1 inhibitors therapy [24, 25]. Identifying predictive biomarkers to distinguish patients most likely to respond to immunotherapy from overall individuals would decrease treatment cost and avoid immune-related adverse events.

## Tumor microenvironment related biomarkers

A possible mechanism of tumor immune escape is adaptive immune resistance, indicating the feedback that IFN-γ-induced upregulation of PD-1/PD-L1 axis could downregulate the cytokines and suppress the immune response in tumor microenvironment [26, 27]. Tumor regression induced by PD-1/PD-L1 inhibitors is influenced by some tumor microenvironment related factors such as PD-L1 status and pre-existing tumor infiltrating lymphocyte (TIL) [13, 26].

### PD-L1 expression

#### Relationship between PD-L1 expression and therapeutic response rate

As the most widely adopted predictor, the role of PD-L1 expression has been investigated in many clinical trials (Table 1). Status of PD-L1 expression (positive/negative) is measured by proportion of PD-L1 expressing tumor cell (TC) and/or immune cell (IC). However, the conclusions from multiple trials are not consistent. Generally believed, high PD-L1 expression is related to increased response rate and clinical benefit in anti-PD-1/anti-PD-L1 therapy [28, 29]. In the phase 2 study Keynote-052, patients with urothelial cancer were treated with pembrolizumab, and increased positive predictive value was obtained along with increased PD-L1 expression cutoff value in the range of 1–10% [30]. And the subgroup with PD-L1 expression above 10% showed higher objective response rate than subgroup with PD-L1 expression below 1% (39% vs. 11%) [30]. However, the correlation between elevated PD-L1 expression and higher response rate is overthrown in some trials. In the study Checkmate-032 which involved patients with urothelial cancer, no significant difference in objective response rate (24.0% vs. 26.2%) was observed between PD-L1 expression positive subgroup (≥1%) and negative subgroup (< 1%) [31].

**Table 1:** Clinical trials of PD-1/PD-L1 inhibitors

Agents	Tumors	PD-L1 IHC platforms	Cells scored by IHC	Cutoff	Efficacy of agent	Clinical trial	Ref.
Pembrolizumab	Urothelial cancer	Dako 22C3 pharmDx Assay	Combined score of TC and IC	< 1%:	11, 95%CI 4–24% (ORR^a^)	Keynote-052	[30]
				1–9%	20, 95%CI 14–28%(ORR^a^)		
				≥10%	39, 95% CI 28–50% (ORR^a^)		
	Melanoma	Dako 22C3 pharmDx Assay	Combined score of TC and IC	< 1%:	36.4, 95% CI 10.9–69.2% (ORR^b^)	Keynote-041	[116]
				≥1%	16.7%, 95% CI 3.6–41.4% (ORR^b^)		
	HNSCC	Dako 22C3 pharmDx Assay	Combined score of TC and IC	< 50%:	13, 95%CI 7–20% (ORR^b^)	Keynote-055	[117]
				≥50%	27, 95%CI 15–42% (ORR^b^)		
	NSCLC	Dako 22C3 pharmDx Assay	TC	< 1%	8.3, 95% CI 0.2–38.5%(ORR^a^)	Keynote-001	[118]
				1–49%	17.3, 95% CI 8.2–30.3% (ORR^a^)		
				≥50%	51.9, 95% CI 31.9–71.3% (ORR^a^)		
	Melanoma	Dako 22C3 pharmDx Assay	Combined score of TC and IC	< 1%	2.8moths, 95% CI 2.7–2.8 months (PFS) 12.6 months, 95% CI 7.0–18.5 months (OS)	Keynote-001	[119]
				≥1%	5.6 months, 95% CI 4.4–8.1 months (PFS) 29.9 months, 95% CI 24.6-NR months (OS)		
Nivolumab	Squamous NSCLC	Dako 28–8 pharmDx Assay	TC	< 1%	HR of 2 years OS between Nivolumab and Docetaxel HR:0.57, 95%CI 0.38–0.86	Checkmate-017	[120]
				≥1%	HR:0.75, 95%CI 0.50–1.10		
				≥5%	HR:0.57, 95%CI 0.36–0.92		
				≥10%	HR:0.56, 95%CI 0.33–0.94		
				≥50%	HR:0.63, 95%CI 0.25–1.57		
	Non-squamous NSCLC	Dako 28–8 pharmDx Assay	TC	< 1%	HR of 2 years OS between Nivolumab and Docetaxel HR:0.91, 95%CI 0.67–1.22	Checkmate-057	[120]
				≥1%	HR:0.62, 95%CI 0.47–0.83		
				≥5%	HR:0.48, 95%CI 0.34–0.68		
				≥10%	HR:0.43, 95%CI 0.30–0.62		
				≥50%	HR:0.38, 95%CI 0.24–0.60		
	Urothelial cancer	Dako 28–8 pharmDx Assay	TC	< 1%	16.1, 95% CI 10.5–23.1% (ORR^a^)	Checkmate-275	[121]
				≥1%	23.8, 95% CI 16.5–32.3% (ORR^a^)		
				≥5%	28.4, 95% CI 18.9–39.5% (ORR^a^)		
	Urothelial cancer	Dako 28–8 pharmDx Assay	TC	< 1%	26.2%; 95% CI 13.9–42.0%(ORR^a^)9·9 months, 95% CI 7.0–not estimable (median OS)	Checkmate-032	[31]
				≥1%	24.0%; 95% CI 9.4–45.1% (ORR^a^)16.2 months, 95% CI 7.6–NE (median OS)		
	Renal cell cancer	Dako Assay^c^	TC	< 1%	HR of median OS between Nivolumab and everolimusHR: 0.76; 95% CI 0.60–0.97	Checkmate-025	[122]
				≥1%	HR: 0.78; 95% CI 0.53–1.16		
	Squamous NSCLC	Dako Assay^c^	TC	< 5%	Best overall response 14% (PR),20% (SD),49% (PD)	Checkmate-063	[123]
				≥5%	Best overall response 24% (PR),24% (SD),44% (PD)		
	Renal cell cancer	Dako 28–8 pharmDx Assay	TC	< 5%	18% (ORR^a^)2.9 months (median PFS)	NCT01354431.	[124]
				≥5%	31% (ORR^a^)4.9 months (median PFS)		
	Melanoma	Dako Assay^c^	TC	< 5% or undefined	ORR^a^: 33.1, 95% CI 25.2–41.7% vs. 15.7%, 95% CI 10.0–23.0% (nivolumab vs. dacarbazine)	Checkmate-066	[125]
				≥5%	ORR^a^: 52.7, 95% CI 40.8–64.3% vs. 10.8%, 95% CI 4.8–20.2% (nivolumab vs. dacarbazine)		
	Multiple cancers	IHC staining with anti-PD-L1 mAb 5H1	TC	< 5%	0% (ORR^a^)	NCT00730639	[126]
				≥5%	36% (ORR^a^)		
Atezolizumab	NSCLC	Ventana SP142 assay	TC or IC	TC and IC < 1%	HR of OS between atezolizumab and docetaxel HR:0.75, 95% CI 0.59–0.96	OAK	[127]
				TC or IC ≥ 1%	HR:0.74, 95% CI 0.58–0.93		
	Urothelial cancer	Ventana SP142 assay	IC	< 1%	21, 95%CI 9–37% (ORR^a^)	NCT02108652	[128]
				1–4%	21, 95%CI 11–35%(ORR^a^)		
				≥5%	28, 95%CI 14–47% (ORR^a^)		
	Renal cell cancer	Ventana SP142 assay	IC	< 1%	9, 95%CI 1–29% (ORR^a^)51, 95%CI 27–74% (2-Years OS Rate)	NCT01375842	[129]
				≥1%	18, 95%CI7–35% (ORR^a^)65, 95%CI45–86% (2-Years OS Rate)		
	Multiple cancers	Ventana SP142 assay	IC	< 1%	13%(ORR^a^), 24-weeks PFS:33.9%	NCT01375842	[130]
				1–4%	21% (ORR^a^), 24-weeks PFS:40.9%		
				5–9%	17% (ORR^a^), 24-weeks PFS:43.0%		
				≥10%	46% (ORR^a^), 24-weeks PFS:60.0%		
	NSCLC	Ventana SP142 assay	TC or IC	TC and IC < 1%	HR of OS between atezolizumab and docetaxel:1.04, 95%CI 0.62–1.75	POPLAR	[131]
				TC or IC ≥1%	HR of OS between atezolizumab and docetaxel: 0.59, 95%CI 0.40–0.85		
				TC or IC ≥5%	HR of OS between atezolizumab and docetaxel: 0.54, 95%CI 0.33–0.89		
				TC ≥50% or IC ≥10%	HR of OS between atezolizumab and docetaxel: 0.49, 95%CI 0.22–1.07		
Durvalumab	Urothelial cancer	Ventana SP263 assay	TC or IC	TC and IC < 25%	5.1, 95%CI 1.4–12.5%(ORR^a^)	NCT01693562	[132]
				TC or IC ≥25%	27.6,95%CI 19.0–37.5%(ORR^a^)		
Avelumab	Urothelial cancer	Dako assay	TC	< 5%	4.2% (ORR^b^)12 months-OS rate: 56.3, 95%CI 33.7–73.9%	NCT01772004	[133]
				≥5%	53.8% (ORR^b^)12 months-OS rate: 75.5, 95%CI 41.6–91.4%		

Abbreviations: *CI* confidence interval, *HNSCC* head and neck squamous cell carcinoma, *HR* hazard ratio, *IC* tumor infiltrating immune cell, *NE* not estimable, *ORR*^a^ objective response rate, *ORR*^b^ overall response rate, *OS* overall survival, *mAb* monoclonal antibody, *PD*, progressive disease, *PFS* progressive-free-survival, *PR* partially response, *SD* stably disease, *TC* tumor cell, *Dako Assay*^c^ anti-PD-L1 antibody is not given

Many hypotheses have been put forward to explain the difference. Firstly, as the immunohistochemistry (IHC) is widely adopted in detection of PD-L1 expression, different cutoff values and scoring systems are used in separate clinical trials [24, 32]. And different antibodies and IHC platforms lead to the incomparability of results among trials as well [33]. Moreover, upregulated PD-L1 expression could be attributed to multiple causes. Intracellular oncogenic variations such as loss of *PTEN* and exposure to TIL-derived cytokines both contribute to upregulated PD-L1 expression [34]. However, immunity dependent PD-L1 upregulation is more meaningful to reactivate the tumor killing activity of TIL while intracellular oncogenic signaling pathway mediated upregulated PD-L1 has limited predictive value [34]. Lastly, due to intratumoral heterogeneity and dynamic alteration of PD-L1 expression along with treatment and cancer progression, the actual status of PD-L1 would be misinterpreted [35, 36].

#### The predictive value of PD-L1 expression in combination therapy

In spite of many limitations mentioned above, PD-L1 status is still a core predictor of treatment effect. However, this viewpoint is challenged in the context of combination strategy. A recent clinical trial interrogated the efficacy of combination strategy including atezolizumab, bevacizumab, carboplatin, and paclitaxel (ABCP) in metastatic non-squamous NSCLC patients [37]. Prognosis of patients receiving ABCP was improved significantly compared with treatment consisting of bevacizumab, carboplatin, and paclitaxel (BCP) [37]. Notably, for patients without epidermal growth factor receptor (EGFR) or anaplastic lymphoma kinase (ALK) variations, ABCP group had prolonged RFS (HR = 0.77, *p* < 0.05, in PD-L1^−^ patients) and OS (HR = 0.78, *p* = 0.02, in PD-L1^−^ and PD-L1^+^ patients) regardless of PD-L1 status in comparison with BCP group [37]. Due to enhanced migration of neoantigen specific T cell and attenuated immune suppression caused by anti-angiogenesis and other treatments, it is difficult to predict alteration of immune microenvironment of PD-L1^−^ patient post combination treatment [38, 39]. Therefore, in the context of combination of multiple drugs, the predictive value of PD-L1 expression is vague and deserves further investigation.

#### The heterogeneity of PD-L1 expression

Heterogeneous distribution of PD-L1^+^ tumor or stromal cell results in discordance between biopsy specimen and resection tissue [40]. Therefore, when resection tissue is not available, especially for some advanced cancer patients, PD-L1 expression of the whole tumor microenvironment might be displayed inaccurately [40, 41]. In the meanwhile, the probability of false negative event is increased. Notably, multiple cores biopsy showed higher sensitivity for selection of PD-L1^+^ patients compared with single core biopsy [40]. Besides, expression of PD-L1 variates during cancer evolution and treatment which is another obstacle to profiling immune microenvironment landscape. Kelly RJ et al. found that a significant shift from PD-L1^−^ to PD-L1^+^ status in 50% advanced esophageal adenocarcinoma patients post chemo-radiation (OR = 6.5, *p* < 0.01) [42]. Tumor immune environment is subject to the influence of multiple factors, which determines the balance of immune surveillance and tolerance status.

### TIL

TIL is a vital component influencing tumor immune microenvironment. Furthermore, TIL density has been confirmed to associate with adaptive upregulation of PD-L1 and clinical benefits [43]. Pre-existing TIL is unleashed by PD-1/PD-L1 inhibitors and then contributes to tumor regression [44, 45]. Recently, a tumor immune microenvironment model which consists of TIL status (presence or absence) and PD-L1 expression status (positive or negative) is established for immunotherapy prediction [46]. Cancer patients are classified into four types in the model, and Type I (PD-L1^+^TIL^+^) tumor is most likely to respond to PD-1/PD-L1 blockade therapy [46]. However, Type III (PD-L1^+^TIL^−^) tumor is prone to resist to monotherapy of PD-1/PD-L1 inhibitors while the combination of PD-1/PD-L1 inhibitors and adjuvant therapy recruiting T cell into tumor bed would help to reverse the resistance [46]. CD8^+^ TIL is believed to be a vital player in killing tumor cell directly and maintaining the immune surveillance which could be spoilt by the signaling produced by PD-1/PD-L1 axis [47]. Solomon B et al. found that high density of CD8^+^ TIL was related with prolonged OS (HR: 0.4, 95%CI: 0.2–0.9, *p* = 0.017) [47].

**Fig. 1:**
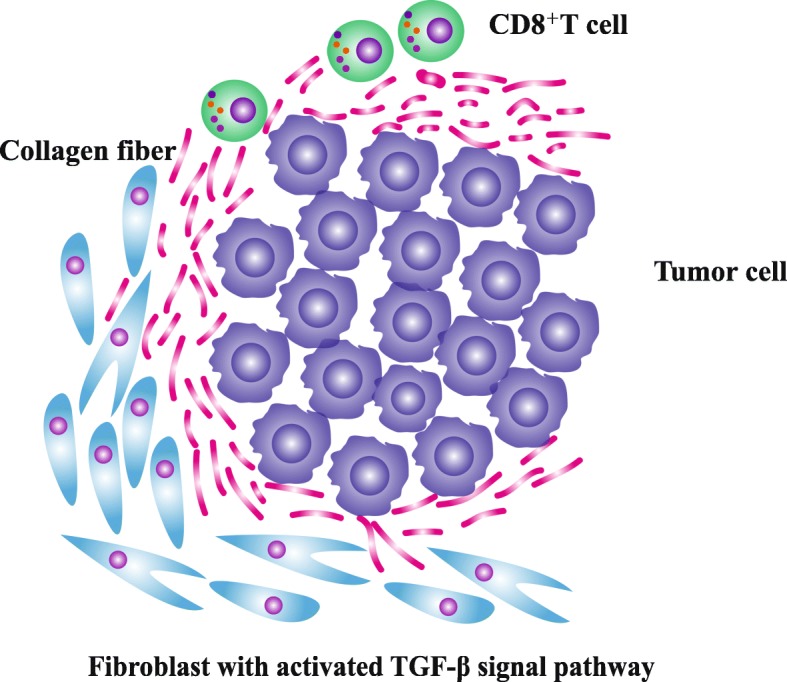
The effect of TGF-β signaling pathway in fibroblast on T cell infiltration. Activated TGF-β signaling pathway in peritumoral fibroblast induces the production of collagen fiber. Collagen fiber surrounding tumor limits T cell infiltration into tumor bed

Simultaneously, in another model based on the status of TIL, tumor immune microenvironment is classified into three subtypes: immune inflamed subtype, excluded infiltrate subtype, and immune ignorance subtype [48]. Recently, transforming growth factor β (TGF-β) signaling pathway attracts extensive attention because of its influence on T cell infiltration and distribution in tumor bed [49–51]. Mariathasan S et al. conducted a study which enrolled metastatic urothelial cancer patients receiving atezolizumab treatment [49]. In the study, it was noticed that infiltration of T cell into tumor bed might be hampered by activated TGF-β signaling pathway in peritumoral fibroblast (Fig. 1) [49]. And simultaneously, tumor-specific T cell tended to distribute in peritumoral stroma rather than in intratumoral parenchyma [49]. The combined application of TGF-β signaling pathway blockade and PD-1/PD-L1 blockade had the significant advantage in tumor control with conversion of tumor environment from excluded infiltrate subtype to immune inflamed subtype [49–51]. Notably, high pan fibroblast TGF-β response signature (TGF-β, TGF-β receptor, etc.) is related with non-response and tumor progression, especially for patient belonging to excluded infiltrate subtype [49].

### TIL derived interferon-γ (IFN-γ)

**Fig. 2:**
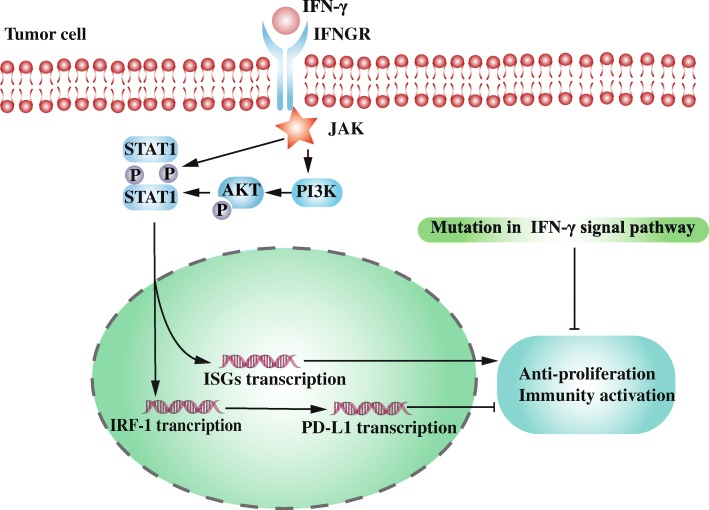
The role of IFN-γ signaling pathway in adaptive immune resistance and immune surveillance. IFN-γ binds to IFN-γ receptor (IFNGR) on the tumor cell membrane and then activates associated Janus kinase (JAK). Subsequent recruitment and phosphorylation of signal transducers and activators of transcription 1 (STAT1) regulate transcription of Interferon Regulatory Factor-1(IRF-1) in nucleus. IRF-1 promotes PD-L1 expression while interferon-stimulated gene (ISG) transcription induced by phosphorylated STAT1 enhances immune response and inhibits tumor proliferation. Phosphoinositide 3-kinase (PI3K)-AKT pathway promotes activation of STAT1. Constant exposure to IFN-γ by anti-PD-1/PD-L1 results in survival selective pressure. Accumulated IFN-γ signaling pathway mutation or epigenetic alteration abrogates CD8^+^ T cell mediated tumor cytotoxicity

IFN-γ signaling pathway is a double-edged sword in immune surveillance. On the one hand, CD8^+^ T cell inhibits tumor cell proliferation and enhances immune activity by secreting IFN-γ. On the other hand, T cell-derived IFN-γ upregulates PD-L1 expression on tumor cell as a shield to protect tumor cells from the immune surveillance’s attack [52, 53]. Upregulated PD-L1 driven by IFN-γ is the hallmark of potential tumor killing activation which is corresponded to Type I (PD-L1^+^TIL^+^) tumor above-mentioned. IFN-γ expression is generally believed to predict a favorable immune microenvironment to anti-PD-1/PD-L1 therapy [54]. *IFNG* mRNA expression extracted from formalin-fixed paraffin-embedded tissue specimens is positively related with the effect of anti-PD-1/PD-L1 treatment [55]. However, with PD-1/PD-L1 blockade, constant exposure to IFN-γ leads to survival selective pressure that tumor cells with defect in IFN-γ signaling pathway are most likely to proliferate (Fig. 2) [56]. Loss of downstream signals of IFN-γ is related to adaptive drug resistance during immunotherapy [52]. As a consequence, intact IFN-γ signaling pathway is a necessary but non-sufficient determinant for robust anti-tumor effect.

In fact, apart from IFN-γ, other inflammatory cytokines could induce adaptive immune resistance in multiple cancers. Tumor necrosis factor-α (TNF-α) mediates the de-differentiation of melanoma cell [13]. Moreover, TNF-α, Interleukin-6 (IL-6), and TGF-β are related to epithelial-to-mesenchymal transition (EMT) in multiple cancers such as melanoma and breast cancer [57, 58]. Notably, the cross-talk between TGFβ/TGFβRII pathway and PD-1/PD-L1 axis has been verified to contribute to T cell anergy in transplantation tolerance, but the mechanism should be investigated in tumor immune microenvironment further [59].

## Tumor intrinsic feature related biomarkers

### Tumor mutational burden

**Fig. 3:**
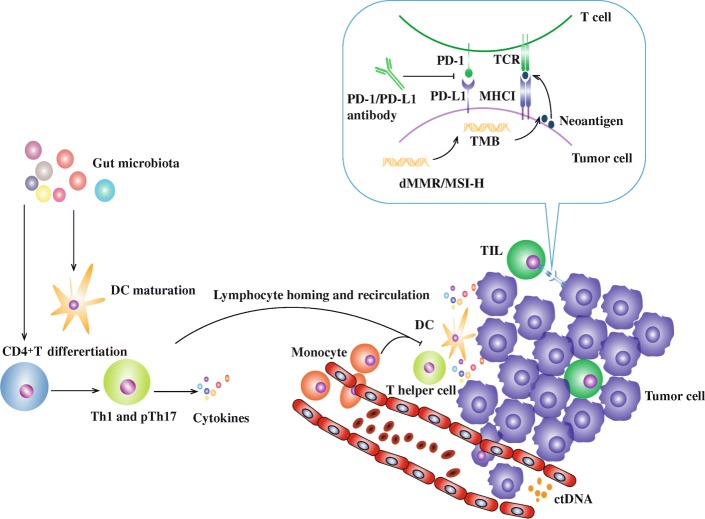
Mechanisms of main biomarkers predicting efficacy of PD-1/PD-L1 inhibitors. Firstly, PD-L1 status reflects adaptive immune resistance which is therapeutic target of PD-1/PD-L1 inhibitors. Mismatch repair deficiency (dMMR) and high microsatellite instability (MSI-H) correlates strongly with high tumor mutational burden (TMB). In the meanwhile, TMB enhances the immunogenicity. Thirdly, tumor infiltrating lymphocyte (TIL) represents potential immune surveillance which could be reactivated by agents. Specific gut microbiota promotes differentiation of T cell, as well as lymphocyte homing and recirculation. Besides, peripheral CD14^+^CD16^−^HLA-DR^hi^ monocyte promotes migration of T cell to tumor bed. Lastly, variation of circulating tumor DNA (ctDNA) and PD-L1^+^ circulating tumor cell presents effect of agent in early stage

As a biomarker independent of PD-L1 expression, accumulated mutations with increased potentiality of neoantigen results in elevated immunogenicity (Fig. 3) [60, 61]. Correspondingly, activated immune microenvironment is favorable to tumor shrink in the context of anti-PD-1/PD-L1 treatment [62]. Based on Next-Generation Sequencing, it is available to profile nonsynonymous somatic mutations of tumor cell [63]. The level of tumor mutational burden (TMB) is evaluated by mutations per megabase [60]. A pooled analysis involving 27 tumor types/subtypes revealed a significant correlation between TMB and objective response rate (correlation coefficient: 0.74) [64]. Notably, clonal mutations (shared by all tumor cells) and subclonal mutations (expressing on a fraction of tumor cells) affect tumor specific immunity differently [65]. McGranahan N et al. found that homogeneous tumor with high TMB associated with increased clinical benefits and sensitivity to anti-PD-1/PD-L1 therapy [65]. However, tumor with high subclonal mutation rate tends to accompany poor anti-PD-1/PD-L1 effect [60]. Single-site biopsy might overestimate level of clonal mutation due to the interference from subclonal mutation which might explain the poor response of some patients with high TMB [62, 65].

### Mismatch repair deficiency and microsatellite instability

Mismatch repair (MMR) system participates in rectifying base-base mismatch, insertion, and deletion defect during DNA replication [66]. Members belonging to MMR system including MutL homolog 1 (MLH1), MutS protein homolog 2 (MSH2), MutS homolog 6 (MSH6), and PMS1 homolog 2 (PMS2) contribute to maintaining genomic stability while reduction or depletion of MMR promotes oncogenesis, especially in gastrointestinal cancers [63, 67]. Mismatch repair deficiency (dMMR) leads to the accumulation of mutation as well as production of potential neoantigen (Fig. 3). Furthermore, MMR IHC and microsatellite instability (MSI) analyzed by Polymerase Chain Reaction (PCR) revealed a high concordance between dMMR and MSI [68]. In fact, the primary reason of MSI is epigenetic or genetic variation of MMR [69, 70]. Xiao X et al. found existence of MSI in all ovarian cancer patients with dMMR [71]. MSI-high (MSI-H)/dMMR associates with favorable prognosis of patients receiving anti-PD-1/PD-L1 therapy [72]. Kumar R et al. observed that anti-PD-1 promoted dMMR tumor cell apoptosis by cytotoxicity of CD8^+^ T cell in vitro in comparison with MMR proficient tumor cell [73]. Le DT et al. conducted a study to explore the influence of MSI-H on anti-PD-L1 therapy, and satisfactory treatment effect was observed (objective radiographic response rate: 53%, complete response rate: 21%) in multiple cancer patients with dMMR [74]. Enhanced treatment effect resulting from MSI-H/dMMR is attributed to increased density of TIL, elevated TMB, upregulated PD-L1 expression, and more potent tumor-specific immune response [72, 75, 76].

## Oncogenic driver mutations and other mutations

It has been found that some driver mutations affect PD-L1 expression such as mutation of *EGFR*, Kirsten rat sarcoma viral oncogene homology (*KRAS*), and *ALK* [77]. *EGFR* activating mutation (*mEGFR*) upregulates PD-L1 expression and impedes the activation of TIL [78]. Contrary to expectation, patients harboring *mEGFR* tends to have poorer response in comparison with patients with wild *EGFR* during anti-PD-1/PD-L1 therapy. PD-L1 expression could be regulated by both extracellular immune factor and intracellular oncogenic driver signal. Given the activated *EGFR*-mediated PD-L1 expression by PI3K-AKT-STAT3/mTOR signaling pathways as well as simultaneous *mEGFR*-induced IFN-γ decline, it is hard to estimate whether PD-L1 expression is regulated just depending on *EGFR* status [78, 79]. Besides, *mEGFR* is relevant to low TMB and compromised tumor-specific immune response [78]. In contrast to *mEGFR*, meta-analysis revealed that NSCLC patients harboring *KRAS* mutation are more likely to belong to PD-L1 positive subtype [80]. And Coelho MA et al. found that hyperactive *KRAS* enhanced stability of PD-L1 mRNA by MEK-ERK signal pathway [81]. Notably, co-occurring mutation with mutated *KRAS* affects tumor microenvironment in different ways. Mutated *KRAS* with co-occurring serine/threonine kinase 11/liver kinase B1 variation associates with upregulated expression of PD-L1 while co-occurring mutation with *TP53* accompanies high TMB abundance [81]. Moreover, *ALK* arrangement in inflammatory myofibroblastic tumor is related to decreased CD8^+^ TIL as well as downregulated PD-L1 expression [82]. Except for driver mutations, some other somatic mutations modulate tumor-specific immune response as well. *Kataegis* is a special mutation pattern which is caused by variation of apolipoprotein B mRNA-editing enzyme catalytic polypeptide-like 3 (APOBEC3) [83]. Boichard A et al. found that *Kataegis* and APOBEC3 overexpression participated in regulation of PD-L1 expression [83]. Furthermore, polymerase δ1 (*POLD1*) and polymerase ε (*POLE*) variations lead to extremely high frequency of somatic mutation which affects tumor immunogenicity [84, 85].

## Gut microbiota

Cross-talk between gut microbiota and host immunity influences anti-tumor effect of anti-PD-1/PD-L1 therapy and the predictive value of gut microbiota has been noticed recently (Table 2) [86]. Using mouse xenograft model, Ayelet Sivan et al. observed that fecal microbiome transplantation could restore the sensitivity to anti-PD-L1 treatment and improve anti-tumor activity in non-responding mice [87]. And increased *Bifidobacterium* abundance accounts for the alteration mentioned above [87]. Besides, Gopalakrishnan V et al. noticed the relationship between high abundance of *Faecalibacterium* genus and elevated response rate in patients receiving anti-PD-1 treatment [88]. In the meanwhile, dysbacteriosis caused by utilization of antibiotics was proved to influence the efficacy of anti-PD-1/PD-L1 therapy. And the poor response to agents could be reversed by recolonization of *Akkermansia muciniphila* [89]. Though the exact modulatory mechanism is unclear, many factors are proposed to enhanced tumor control (Fig. 3). Firstly, *Bifidobacterium* promotes maturation and activation of dendritic cell (DC) which enhances neoantigen presentation process [87]. Secondly, recolonization of *Akkermansia muciniphila* and *Enterococcus hirae* associates with appearance of CD4^+^ central memory T cell (T_CM_) in tumor bed [89]. And T_CM_ leads to increased CD4/Foxp3^+^ ratio in tumor bed by enhancing recruitment and chemotactic migration of T cell [89]. Thirdly, bacteria could be sensed by host immunity and then influences the differentiation of lymphocytes such as Th1 and pTh17 in second immune organ. The alteration of microbiota composition might change the tumor immune microenvironment by the homing and recirculation of lymphocytes [89, 90]. Furthermore, bacterial metabolites such as short chain fatty acid (SCFA) participates in energy metabolism of immune cell which might affects the function of immunity [91]. Finally, potential molecular mimicry between gut microbiota and tumor might participates in tumor-specific immune response [92]. Therefore, analyzing gut microbiota composition would be favorable to predict treatment effect of anti-PD-1/PD-L1 therapy.

**Table 2:** The role of gut microbiota in PD-1/PD-L1 inhibitors therapy

Bacteria	Main effect on immunity	Prediction of treatment effect	Model	Ref.
*A. muciniphila*	Increased recruitment of CCR9^+^CXCR3^+^CD4^+^ T cells into tumor bed	Effective anti-tumor response	Mouse/Human	[89]
*E. hirae*	Increased IL-12 secreted by DC	Effective anti-tumor response	Mouse/Human	[89]
*E. faeciumC. aerofaciens* *B. adolescentisK. pneumoniae* *V. parvulaP. merdae* *Lactobacillus sp. B. longum*	Increased neoantigen specific CD8^+^ T cell and decreased Fox3P^+^CD4^+^ Treg in tumor microenvironment	Effective anti-tumor response	Mouse/Human	[134]
*Bifidobacterium*	Increased IFN-γ production and major histocompatibility complex Class II^hi^ DC	Effective anti-tumor response	Mouse	[87]
*Faecalibacterium*	Increased peripheral effector CD4^+^ and CD8^+^ T cell	Effective anti-tumor response	Mouse/Human	[88]
*Bacteroidales*	Increased peripheral Treg and myeloid derived suppressor cell	Poor anti-tumor response	Mouse/Human	[88]

Abbreviations: *IFN-γ* interferon-γ, *Treg* regulatory T cell, *DC* dendritic cell

## Biomarkers in peripheral blood

Compared with biopsy sample from tumor tissue, peripheral blood sample is more available and less heterogeneous. Due to negligible invasion, it is an ideal access to monitor shift of biomarkers in peripheral blood for optimized therapy strategy (Fig. 3) [93].

### Peripheral immune cell

Using mass cytometry and bioinformatics analysis, Krieg C et al. observed that high abundance of peripheral CD14^+^CD16^−^HLA-DR^hi^ monocyte at baseline associated with higher response rate in anti-PD-1/PD-L1 therapy. And the increased markers on membrane such as intercellular cell adhesion molecule-1 (ICAM-1) and human leukocyte antigen-antigen D related (HLA-DR) indicate enhanced migration and activation of monocyte. Besides, responding patients tended to have decreased T cell in peripheral blood in comparison with non-responding patients. Supposedly, CD14^+^CD16^−^HLA-DR^hi^ monocyte promotes the infiltration of T cell from peripheral blood into tumor bed which results in enhanced T cell-mediated tumor killing activity [94]. Besides, Kamphorst AO et al. noticed that early expansion of peripheral PD-1^+^Ki-67^+^CD8^+^ T cells after anti-PD-1 treatment was related to better treatment effect. And peripheral PD-1^+^Ki-67^+^CD8^+^ T cell was detected to express more activation-associated markers such as cytotoxic T-lymphocyte-associated protein 4 (CTLA-4) and inducible T cell costimulator (ICOS) [95]. Furthermore, Fujisawa Y et al. found that neutrophil/lymphocyte ratio and lactate dehydrogenase (LDH) level associated with response to nivolumab in melanoma patients. Elevated neutrophil/lymphocyte ratio (> 2.2) predicted poor treatment effect (OR = 4.16, *p* = 0.0026) while increased peripheral LDH was related with poor response tendency without statistical significance (OR = 2.53, *p* = 0.081) [96]. Contrary to neutrophil, increased relative eosinophil count (≥1.5%) could be used as a favorable predictor in melanoma patients receiving pembrolizumab [97].

### Circulating tumor DNA and PD-L1^high^ circulating tumor cell

Radiological assessment is widely applied to evaluate the treatment effect of anti-PD-1/PD-L1. However, interference of pseudo-progression and non-real time reflection of tumor burden might affect the selection of subsequent treatment strategy [98]. It was observed that circulating tumor DNA (ctDNA) in responding patient decreased quickly in 5 days after first nivolumab administration. The phenomenon is meaningful that the shift in ctDNA is prior to second administration and radiological change [99]. Compared with detectable abundance at baseline, undetectable ctDNA after therapy beginning indicates robust anti-tumor effect which is valuable for early patient selection [98, 100]. Similarly, decreased PD-L1^+^ circulating tumor cell after treatment beginning is related to robust anti-tumor response. However, patients with high abundance of PD-L1^+^ circulating tumor cell at baseline tend to be sensitive to anti-PD-L1 therapy [101].

### Soluble PD-L1

Splice variants of PD-L1 which lack transmembrane or intracellular domain lead to secretion of soluble PD-L1 (sPD-L1) [102]. Similar to membrane-binding PD-L1, sPD-L1 hampers the activation and proliferation of T cell as well [103]. It is generally acknowledged that increased level of sPD-L1 before treatment associates with poor prognosis which is attributed to high tumor burden, elevated alternative splicing, and exhausted immune response [102, 104]. Zhou J et al. found that high sPD-L1 at baseline was related with increased risk of tumor progression. However, rapidly increased sPD-L1 level after immune checkpoint inhibitors treatment indicated potent tumor-specific immune response and high partial response rate (around 70%) [102].

### Peripheral cytokine and other parameters

Peripheral cytokines reflect status of tumor immune microenvironment and response to anti-PD-1/PD-L1 treatment [93]. Prolactin (PRL) participates in maturation and activation of immunity while high PRL inhibits immune response by IL-10 [105]. Adaptive hyperprolactinemia associates with poor response during nivolumab treatment and patients with stable concentration of PRL exhibit significant higher response rate (*p* = 0.004) [105]. Moreover, a phase 2 study revealed that pretreatment high level of IFN-γ, IL-6, and IL-10 in peripheral blood were relevant to increased objective response rate in melanoma patients receiving nivolumab [106]. Besides, tumor-derived vascular endothelial growth factor (VEGF) promotes tumor progression by angiogenesis and immunosuppression in tumor microenvironment [107]. Anti-angiogenesis therapy not only inhibits neo-vascular formation, but also upregulates the quantity of TIL significantly [108]. Patients receiving anti-PD-L1 combined with anti-VEGF therapy exhibited higher response rate than monotherapy [107, 109]. Cytokines participate in immune response directly, and the predictive value of cytokine in peripheral blood needs to explore further.

## Patient previous history, pathological feature, and other predictors

Chronic obstructive pulmonary disease (COPD) participates in oncogenesis and COPD-associated chronic inflammation influences immune environment of lung cancer patient in the meanwhile [34]. Biton J et al. interrogated treatment response of lung cancer patients receiving nivolumab. Lung cancer patients with co-existing COPD tended to harbor higher inhibitory markers such as PD-1 and TIM-3, which indicated more severe exhaustion of TIL in comparison with patients without COPD [34, 110]. NSCLC patients with co-existing COPD had favorable prognosis during nivolumab treatment and increased correlation between PD-L1 expression and response rate [34]. Notably, cigarette exposure contributes to oncogenesis of lung cancer as well as occurrence of COPD [111]. Because cigarette exposure leads to increased TMB which might cause enhanced the sensitivity to immunotherapy, it is necessary to rule out the interference from cigarette exposure [112]. By analyzing TMB, *KRAS*, and *TP53* variations in COPD^+^ patients, no significant enrichment of smoking signature was observed in COPD^+^ patients [34]. Therefore, COPD is speculated as a potential predictor for anti-PD-1/PD-L1 treatment. Besides, immune microenvironment alters among tumors with different pathological features. In three subtypes of lung adenocarcinoma, the level of TMB and immune cell signature change significantly [113]. Tumor belonging to proximal inflammatory subtype tends to have higher TMB, *TP53* variation, and immune cell signature, while tumor belonging to terminal respiratory unit subtype is most likely to harbor low TMB without *TP53* mutation [113]. And the predictive value of pathological feature needs to be verified in large sample size. Intriguingly, a recent pilot study revealed the correlation between family history of cancer and treatment effect of anti-PD-1/PD-L1 therapy [114]. Multiple cancers patients with family history of cancer had significantly improved objective response rate (*p* = 0.0024) and favorable outcome [114].

## Conclusion

PD-L1 expression is generally believed as a surrogate of pre-existing immune specific immune activity and can be upregulated by IFN-γ in tumor microenvironment [115]. However, other factors simultaneously influence PD-L1 expression such as intracellular oncogenic signaling pathway apart from adaptive immune resistance. Therefore, total PD-L1 including IFN-γ-derived and IFN-γ-independent PD-L1 is not accurate to reflect tumor immune surveillance status [115]. Combination of PD-L1 expression, TIL, TMB, genetic and epigenetic variation of IFN-γ provides a comprehensive prospective on tumor immune landscape. Moreover, circulating biomarkers and gut microbiota play a vital role in dynamic monitoring of tumor immune status due to minimum invasion. With the increased understanding of tumor immune escape, establishing a wide-ranging framework which consists of multiple biomarkers is quite necessary for patient selection and precision medicine.

## Abbreviations

*ALK*: Anaplastic lymphoma kinase
APOBEC3: Apolipoprotein B mRNA-editing enzyme catalytic polypeptide-like 3
COPD: Chronic obstructive pulmonary disease
CTLA-4: Cytotoxic T-lymphocyte-associated protein 4
DC: Dendritic cell
dMMR: Mismatch repair deficiency
EGFR: Epidermal growth factor receptor
EMT: epithelial-to-mesenchymal transition
HR: hazard ratio
IC: Immune cell
ICAM-1: Intercellular cell adhesion molecule-1
ICOS: Inducible T cell costimulator
ITIM: Immune receptor tyrosine based inhibitory motif
ITSM: Immune receptor tyrosine-based switch motif
*KRAS*: Kirsten rat sarcoma viral oncogene homology
LDH: Lactate dehydrogenase
*mEGFR*: *EGFR* activating mutation
MLH1: MutL homolog 1
MMR: Mismatch repair
MSH2: MutS protein homolog 2
MSH6: MutS homolog 6
NSCLC: Non-small-cell-lung cancer
OS: Overcall survival
PD-1/PD-L1: Programmed cell death protein 1/programmed cell death 1 ligand 1
PMS2: PMS1 homolog 2
*POLD1*: Polymerase δ1
*POLE*: polymerase ε
PRL: Prolactin;
SCFA: Short chain fatty acid
SHP1/2: Src homology 2 domain containing phosphatases 1/2
sPD-L1: soluble PD-L1
TC: Tumor cell
T_CM_: Central memory T cell
TGF-β: Transforming growth factor β
TMB: tumor mutational burden
TNF-α: Tumor necrosis factor-α
VEGF: Vascular endothelial growth factor
